# Light Interception, Photosynthetic Performance, and Yield of Oil Palm Interspecific OxG Hybrid (*Elaeis oleifera* (Kunth) Cortés x *Elaeis guineensis* Jacq.) under Three Planting Densities

**DOI:** 10.3390/plants11091166

**Published:** 2022-04-26

**Authors:** Hernán Mauricio Romero, Stephany Guataquira, Diana Carolina Forero

**Affiliations:** 1 Department of Biology, Universidad Nacional de Colombia, Bogotá 11132, Colombia; 2 Colombian Oil Palm Research Center—Cenipalma, Oil Palm Biology and Breeding Research Program, Bogotá 11121, Colombia; sguataquira@cenipalma.org (S.G.); dforer5@gmail.com (D.C.F.)

**Keywords:** planting density, photosynthetically active radiation, extinction coefficient, leaf area index, gas exchange

## Abstract

Environmental conditions are crucial for crops’ growth, development, and productivity. One of the most important physiological factors associated with the production of crops is the use of solar radiation for the photosynthesis process, which determines the amount of assimilates available for crop growth and yield. Three age classes (4, 6, and 14 years) and three planting densities (143, 128, and 115 palms ha^−1^) were evaluated in a commercial interspecific *Elaeis Oleifera* x *Elaeis guineensis* hybrid Coari x La Mé. The light interception patterns and the photosynthetic performance were determined. Measurements were taken of the leaf area, the number of leaves, and incident and photosynthetically transmitted active radiation. Also, photosynthetic rates, light, and yield were measured. The canopy extinction coefficient (*Kc*) was estimated using the Monsi and Saeki model. Under the evaluated conditions, the average *Kc* value for 4-year-old palms was 0.44; for the 6-year-old group of palms, the average value was 0.40, and 0.32 for the 14-year-old palms, with coefficients of determination (R^2^) greater than 0.8. A pattern associated with the age of the crop was observed, where the *Kc* decreased in groups of adult palms. The results showed increased *Kc* as the planting density decreased. No statistically significant differences were observed between planting densities or ages in the light and CO_2_ curves regarding photosynthesis. The leaf level in which the measurement was made influenced photosynthesis. Thus, the highest values of the photosynthesis parameters were observed in leaf 17. The crop yield tended to stabilize 8 years after planting under 143 and 128 palms per hectare, but 14 years after planting, the Fresh fruit bunch (FFB) production was still growing under 115 palms per hectare. The results showed that, up to year 14 after planting, the highest cumulative yield was achieved with 115 palms per hectare. This was partly caused by a sharp decline in production observed under 128 palms per hectare, which could indicate that in the long production cycle of the OxG hybrids, the 115-palms-per-hectare planting density would result in higher cumulative FFB production. Furthermore, the results showed that the optimum planting density for the hybrids of the present study would be 120 palms ha^−1^, corresponding to a planting distance of 9.8 m between plants.

## 1. Introduction

Oil palms are the perennial, tropical crop that produces the highest yield of vegetable oil per area. It is widely grown in the tropical zones of the world [1], mainly in Southeast Asia with 74% of the planted area, followed by Africa with 20% of the area, and America with 5% [2]. The oil palm belongs to the genus *Elaeis*. The two most notable species are the African oil palm (*Elaeis guineensis* Jacq.), which is native to the humid regions of tropical Africa and is currently cultivated in the humid tropics, and the American oil palm (*Elaeis oleifera* (Kunth) Cortés), which grows naturally in the rainforest margins and poorly drained areas in clay soils and savannas from Costa Rica to Northern Brazil [3]. Although these two species have been geographically isolated, they are fully compatible. Thus, fertile, and productive hybrids can be obtained from them [4]. The resulting OxG interspecific hybrids have desirable traits [5,6], such as producing oils with higher unsaturated fatty acid content [7]. Also, the OxG hybrids have partial resistance to diseases, such as bud rot [8,9], which dramatically affects the yield and, in many cases, becomes a lethal disease. Furthermore, the OxG hybrids could yield high amounts of oil. Thus, it has been shown that, with appropriate management, applying auxins, such as naphthalene acetic acid [10], and harvesting at the closest point to optimal ripening [11], it could be possible to obtain more than 10 tons of oil per hectare per year [12] from OxG hybrids.

The yield of a crop such as the oil palm depends on its genotype and the interaction with environmental factors such as radiation, relative humidity, water availability, soil structure, and agronomic management conditions [13,14]. One of the most important physiological factors associated with the production of crops is the use of solar radiation [15], as it stimulates development processes and is the primary source of energy in the photosynthetic process. These processes are initiated by the absorption of light (quanta) and depend on specific photoreceptors in the plant, the spectral composition of the light, the season, and the duration and direction of the incident radiation. Most solar radiation is absorbed by the crop canopy and is intercepted by leaf blades. However, the petioles, stems, and some reproductive structures can contribute to the whole plant’s photosynthesis. Thus, the photosynthetic process in a community of plants is affected by the intensity and spectral distribution of radiation within the crop canopy and the spatial arrangement of the photosynthetically active area [16]. This arrangement is determined by the foliage distribution within the layers of the canopy, the angle between the incident radiation and the leaf, and the incident radiation conditions [17,18].

Oil palms are a highly productive crop under optimal management conditions. Therefore, their dry-matter production is related to the amount of photosynthetically active radiation (PAR) intercepted by the crop canopy [19]. For the study of the radiation distribution in the canopy, models such as the Monsi and Saeki model [20] have been formulated in dense crops or rows that later form a continuous sheet of leaves and are based on the leaf area index (LAI) and the extinction coefficient (*K*) according to the Beer−Lambert law [19]. Thus, the radiation extinguishes exponentially with increasing depth in the canopy. The relationship is given as:

f = 1 – exp(−*K* ∗ LAI) (1)

where f is the interception of radiation by the canopy, *K* is the extinction coefficient, and LAI is the leaf area index. Knowing the LAI and *K*, f can be calculated, or the LAI can be derived by measuring the value of f. However, the measurement of the radiation distribution in the oil palm canopy is difficult to do because as the crop grows, its canopy reaches several meters above the ground. What is measured is the horizontal variation of the radiation below the canopy, which depends on the LAI in different planting densities. Therefore, it is better to refer to the extinction coefficient of light obtained in this way as the canopy extinction coefficient (*Kc*) [21]. In a planting density trial of 10-year-old African oil palms (*Elaeis guineensis* Jacq), Corley et al. [22] used the radiation extinction model (Equation (1)) and found a *Kc* of 0.44. The results found in this study showed a variation in LAI and leaf area distribution values due to the planting densities used. 

Similarly, Squire [23] carried out intensive studies on radiation interception in oil palms and provided a detailed description of the methods used for measuring light transmission in the crop canopy. Later, Breure [24], in an experiment in 14-year-old palms in Papua New Guinea, reported a *Kc* value of 0.32 using a previously described method [25] and applied a constant α = 0.19 for better data adjustment (Equation (2)), similar to Corley [22], who used the variation in LAI in a density trial to find the radiation extinction coefficient for oil palm. The results showed that it is a suitable model for LAI < 2:

f = 1 − exp(−*K*[LA − α]) (2)

where α represents an x-axis intercept in a graphic of ln(1 − f) against LAI [23].

As the structure of the oil palm canopy changes with age, there is a variation in *Kc,* which partly explains the variation in the values obtained by different authors [26].

The LAI is the factor that most affects the ratio of total solar radiation intercepted. However, its non-linear relationship with (f) means that less radiation is intercepted as the LAI increases. Squire [27] estimated that 95% of the incident PAR is intercepted in African oil palm once the LAI exceeds 5.8. 

One of the most critical factors affecting LAI in oil palms is the planting density. It is essential to plant the crop under an adequate density to reach a rapid canopy closure when the plants are young and immature while avoiding light competition between plants when the trees are grown and mature [28]. The effect of the planting density on LAI directly impacts the light interception of the plants, which is related to the photosynthetic performance of plants and is a critical factor in the crop yield. 

Photosynthesis varies with the concentration of CO_2_, the intensity of solar radiation, temperature, concentration and availability of nutrients, and water status of the plant. The photosynthetic capacity of plants is directly related to productivity and yield. The determination of this capacity allows for establishing the points at which light is converted into biochemical energy and biomolecules [29]. The photosynthetic capacity can be evaluated using light and CO_2_ curves in which compensation points and saturation points are determined. The light compensation point is the irradiance at which photosynthesis and respiration are equal, so CO_2_ is not consumed or released from the plant. CO_2_ is not captured through photosynthesis below the compensation point.

On the contrary, CO_2_ is released into the environment through respiration. Therefore, above the light compensation point and as irradiance increases, CO_2_ uptake increases linearly until maximum photosynthesis (Amax) is obtained. This Amax is reached at the light saturation point, representing the irradiance beyond which carbon uptake does not increase [30].

Therefore, the purpose of this work was to determine the effect of the planting density on the yield, photosynthetic behavior, and the patterns of interception of photosynthetically active radiation in an OxG interspecific hybrid cultivar, Coari x La Mé, by calculating the solar radiation extinction coefficients, characterizing the canopy, constructing light curves, and measuring FFB yield in three age classes and planting densities. 

## 2. Results and Discussion

### 2.1. Patterns of Canopy Development

The development of the canopy is an important parameter as it determines the radiation interception. It is mainly characterized by the LAI dependent on leaf area, leaves per palm, and the planting density. The results showed that the number of leaves decreased with age in the three planting densities (Table 1). Between 41 and 47 leaves per palm were recorded in the 4-year-old group of palms. The leaves per palm in the 6-year-old group of palms ranged from 42 to 44 on average, and in the 14-year-old group of palms, it was between 35 and 38. 

It is important to note that high planting densities reduce the number of leaves per palm [22] due to a decrease in the leaf emission rate, related to slow height increment and less leaf initiation. However, under high planting densities, the competition for light induces stem growth [31], related to leaf emission [32]. This effect could be more pronounced in slow growth cultivars, such as in the interspecific OxG hybrids. In the same way, palm age strongly influences leaf emission, with young oil palms producing more leaves than older palms, which, in the present study, is represented by the higher number of leaves found in the canopy. In the present study, 4-year-old palms had 45 leaves, 6-year-old palms had 43 leaves, and 14-year-old palms had 37 leaves on average. The reduction in leaf emission rate reaches the lowest number when it stabilizes at around 2 leaves per month (24 leaves per palm per year) [7,33]. Although the number of leaves does not express leaf emission in our results, it could indicate the persistence of the fronds or their initiation.

The age and the palm density greatly influenced the intercepted radiation, the transmitted radiation, the LAI, and the *Kc* for total and PAR radiation (Table 1). In the case of LAI, the 4-year-old group of palms showed values of 2.7 (115 palms ha^−1^), 2.5 (128 palms ha^−1^), and 2.4 (143 palms ha^−1^). The trend changed in the 6-year-old group of palms, where the highest LAI was found in palms planted at a higher density (143 palms ha^−1^) with a value of 4.3, followed by the group planted at 9.5 m spacing, with a value of 3.7, and 3.0 for palms planted at 10 m. spacing. The 14-year-old group of palms had the highest LAI value of 5.2 in the highest planting density (143 palms ha^−1^). The trends in the results are similar to those obtained by Henson and Dolmat [14] in the Guthrie (DxP) material, where they performed a physiological analysis in a 16-year density trial and showed that the highest densities had the highest LAI values, and the PAR interception was therefore higher. According to Corley [22], high planting densities indirectly affect the LAI by reducing the number of leaves per palm. However, the leaf number was not reduced with the planting density in our case. In all the analyzed plant ages, the lower the density, the lower the number of leaves per palm. This finding and LAI results would indicate that the increased planting density reduces leaf area. In turn, a reduction could have a negative impact on the photosynthetic capacity of the palms, especially when the palms are older and the differences in leaf number are minimal, but the LAI is strongly affected. In this case, the interspecific OxG hybrids behave like many other species. Light competition strongly affects leaf development, resulting in smaller leaves not compensated by the more significant number of leaves counted in the highest density [34].

### 2.2. Radiation Interception

**Figure 1:**
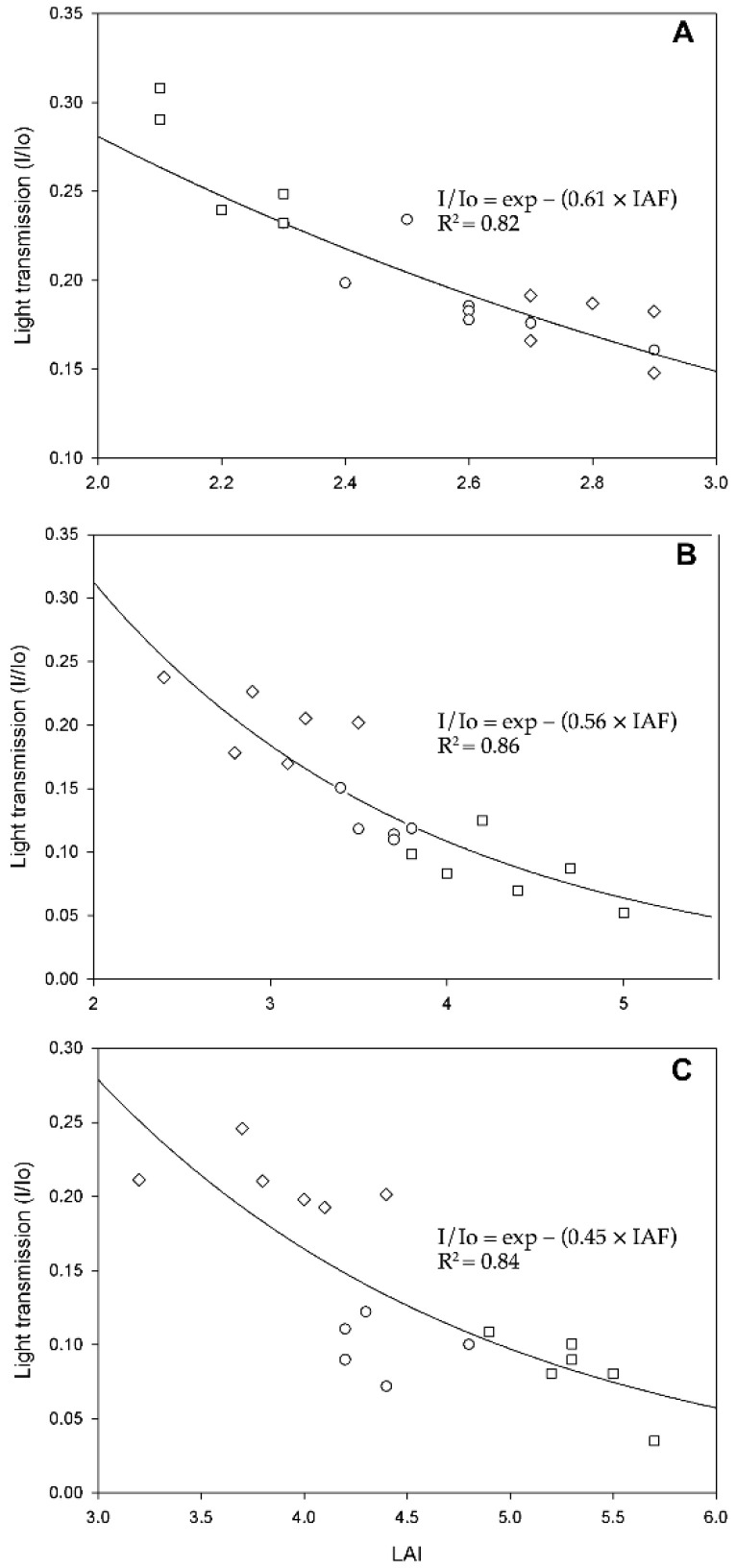
Relationship between transmitted radiation and the leaf area index of palms at three planting densities: (**A**) 4-year-old palms, (**B**) 6-year-old palms, and (**C**) 14-year old palms (*n* = 10). Squares = 143 palms ha^−1^. Circles = 128 palms ha^−1^. Diamonds = 115 palms ha^−1^.

Figure 1 shows the adjusted regression lines for each age group in the evaluated planting densities. The model proposed by Monsi and Saeki (Equation (1)) was used because the adjustment of the equation was better than the model with the intercept used by Squire [23] and Breure [35] (Equation (2)).

In the 4-year-old group, the *Kc*PAR was 0.61, and *Kc*total radiation was 0.44 with a determination coefficient (R^2^) of 0.82. At this time, the crop canopy is not yet closed. Therefore, a lower proportion of intercepted solar radiation and higher transmitted radiation to the lower layers was observed. In this case, 128- and 143-palms-per-hectare densities showed a radiation interception ranging from 76% to 78%. The density of 115 palms per hectare showed a radiation interception of 83%, possibly due to a more significant number of leaves. In this case, the plot already has a closed canopy. As in oil palm, the canopy is considered closed when 80% of the incident radiation is intercepted by the crop [36] or when the neighboring leaves overlap.

In the 6-year-old group of palms, the *Kc*PAR = 0.56 and *Kc*total radiation = 0.40 (total radiation), with an R^2^ = 0.86. This value is higher than what was reported by Gerritsma [21], with a *Kc* = 0.35, and Breure [36], with a value of 0.34 for *Kc*total radiation in *E. guineensis* palms. The intercepted and transmitted PAR shows that the highest planting density (143 palms ha^−1^) has a more significant intercepted radiation (90.5%), while the lowest density (115 palms ha^−1^) intercepts about 79.6% of incident radiation [7].

In general, the 6-year-old palms in the three densities had reached a closed canopy condition, and it is likely that in densities of 128 and 143 palms ha^−1^, the canopy closed earlier. In African oil palms, this situation occurs in densities between 136–143 palms ha^−1^ and about five years after planting. Still, the precise time depends on other factors, such as soil fertility, climate, and the type of planting material [37]. It is important to note that the oil palm canopy closes before the leaves have reached their maximum size, unlike other crops [22]. 

Mature hybrids (14 years old) had a *Kc*PAR of 0.44 and *Kc*total radiation of 0.32 with an R^2^ = 0.85. These values are lower than what is typically found for *E. guineensis,* where values of *Kc* = 0.34 (total radiation) and 0.47 (PAR) are reported [38]. However, Breure [36] found a lower value in 14-year-old palms (*Kc* = 0.32 for the PAR) than in other crops. These values are not that high, so various authors have suggested some causes, such as non-random leaflet distribution [38]; defined phyllotaxis patterns in the formation of new leaves that prevent the crop from filling the gaps in the canopy [19]; and the possible overestimation of the leaf area and therefore of the LAI, resulting in low *Kc* values [39].

As for the PAR interception in this age group, palms planted at 9 m spacing (LAI = 5.2) had the highest values (92%). The plot planted at 9.5 m spacing intercepted about 89% of the radiation; these results are similar to those obtained by Squire [27] for intercepted radiation in 10-year-old *E. guineensis* palms, where he found maximum and minimum values of 96% and 85%, respectively, at a planting density of 148 palms ha^−1^. Finally, palms planted with 10 m spacing had the lowest proportion of intercepted radiation (79%), but a higher proportion of radiation transmitted to the lower layers (20%) was also observed. Also, palms under this density showed the lowest value for *Kc* (0.41). Thus, less radiation is absorbed per unit of LAI, resulting in more PAR available in the lower layers. Low *Kc* values can improve photosynthetic rates under optimal LAI values. In crops where planting density or spacing makes it impossible to have a high LAI, a low *Kc* could mean a low PAR usage. 

*Kc*PAR values obtained for the oil palm interspecific hybrids decrease with age. These values range from 0.61 (at 4 years old) to 0.56 (at 6 years old). These *K* values suggest that the canopy of the OxG hybrids tends to be of a plagiophile-type in young palms, following what was reported by Corley [22]. This situation enables a more uniform light distribution and interception. It reduces the proportion of leaves exposed to radiation saturation levels for photosynthesis in the upper part. It reduces the number of leaves exposed to levels below the light compensation point in the lower layers of the canopy.

In adult palms (14 years old), although higher average LAI values were obtained than in 6-year-old palms, *Kc* radiation interception values at densities of 143 and 128 palms ha^−1^ increased by only 2%. In contrast, at a density of 115 palms ha^−1^, the percentage of intercepted radiation remained almost unchanged. This can be an indicator that, at this time, the canopy expansion is entering the stabilization phase [40]. This work suggests that this stabilization phase occurs later in OxG hybrids than in *Elaeis guineensis,* which, under favorable conditions, usually enter this phase 10 years after planting. This stabilization phase is characterized by the low production of leaves due to the number of leaves pruned for harvesting bunches from very tall palms and lower radiation interception (low *K* values) associated with the change in leaf angles in combination with variations in palm height within the plantation [36]. 

### 2.3. Photosynthesis

In perennial crops, such as oil palm or other forestry species, the capturing of energy and CO_2_ is determined by the total leaf area, the leaf distribution in the canopy, and the photosynthesis of individual leaves. Ultimately, productivity will depend on the photosynthetic performance of the canopy, which, in turn, is determined by the distribution of the sunlight. As can be seen, the *Kc* value is not constant for a crop canopy. It can be affected by factors relating to the sun’s position, the relationship between direct and diffuse radiation, and any change in the structure of the canopy or the leaves due to age or agronomic practices. Therefore, in oil palm plantations, the competition for solar radiation increases with higher planting densities. Although the higher densities result in greater interception of solar radiation (due to an increase in LAI), caution must be exercised as high densities may have a contrary effect. They can reduce dry-matter production per palm due to the increased competition for yield-limiting resources, such as water and nutrients. In other words, maximizing the interception of light through a substantial increase in the leaf area index (LAI) does not necessarily increase yield. 

**Figure 2:**
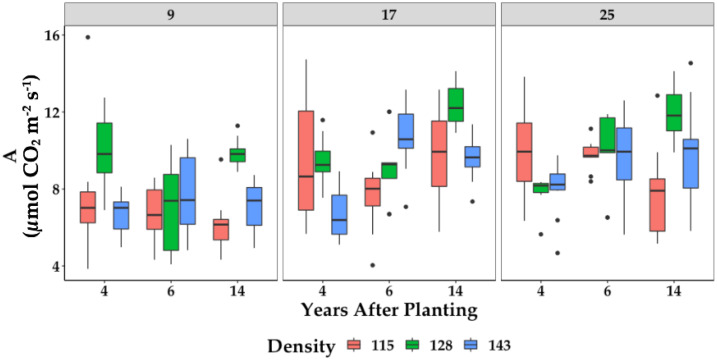
Photosynthetic rate (A) of leaves 9, 17, and 25 in 4-, 6-, and 14-year-old palms planted under three densities (115, 128, and 143 palms ha^−1^). Each box represents the data of leaves from 10 individual palms.

The planting density did not affect the net photosynthetic rate (A) (Figure 2). However, the analysis of variance showed significant differences between the years after planting, the leaves, and their interaction. In general, the photosynthetic rate changes with the age of the plants, which could be related to metabolic changes and physiological responses derived from the aging of the plants [22]. This response is more evident when the photosynthetic rate of individual leaves at different levels in the phyllotaxis (age of the leaf) is analyzed. It has been reported that the photosynthetic rate of the leaves is low in young leaves (Leaf 1) and progressively increases until it reaches the highest values in Leaf 17 and 25. After that, the aging of the leaves results in the reduction of the photosynthesis rate in older leaves (Leaf 33) [34].

**Figure 3:**
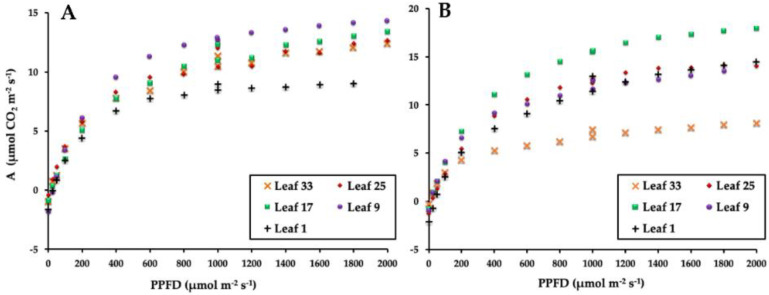
Photosynthesis as a function of irradiance in different age leaves of 6-year-old (**A**) and 14-year-old (**B**) palms. The figures are representative of 90 light response curves made for the experiment. Three different plants were used to construct the light curves for each leaf age, planting density, and age, with at least two repeated measures of the same leaf.

In terms of the photosynthetic response, which in this case measures the highest reachable photosynthesis rate under optimal CO_2_ concentration with different PPFD, the light response curves of varying-age leaves in 6-year-old (Figure 3A) and 14-year-old (Figure 3B) palms show the highest Amax in Leaf 17 in both cases. However, the Amax was higher in the 14-year-old palms than in the 6-year-old palms. In both cases, the photosynthetic capacity was reduced with the age of the leaf. Thus, the lowest photosynthetic response was measured in the 14-year-old palms in Leaf 33. This is per the photosynthetic rate measured (Figure 2) and is related to the phenology of the leaf [41,42]. Leaf 1 is very young and not always fully expanded. From Leaf 9, the metabolic status of the leaves is more stable, and it is generally accepted that the metabolic peak is reached in Leaf 17, with a slight decrease in Leaf 25, but with strong signs of senescence in Leaf 33 [22]. 

**Figure 4:**
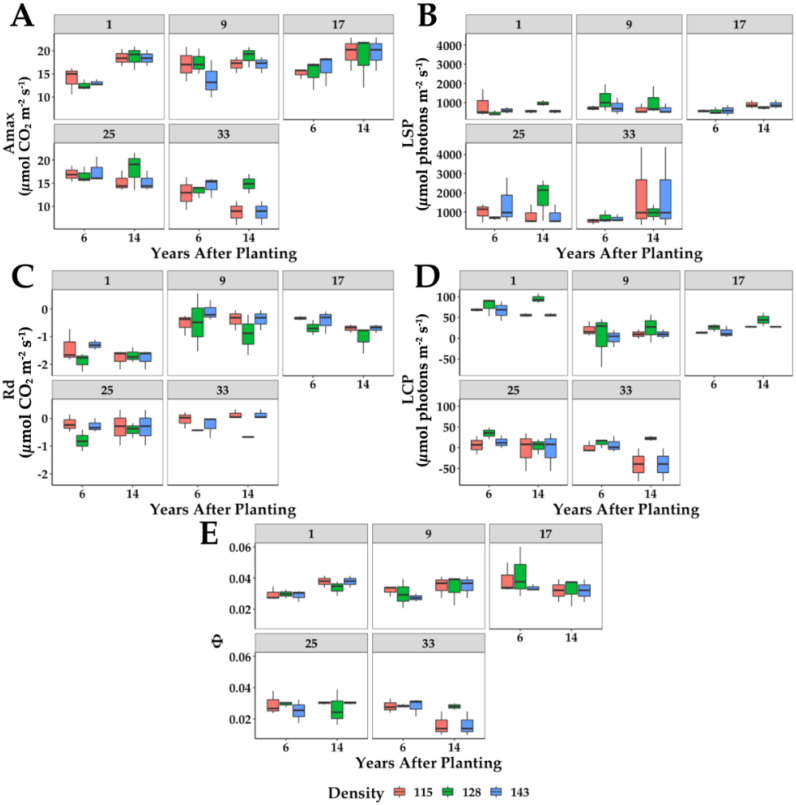
Parameters of photosynthetic performance derived from light response to curves of leaves 1, 9, 17, 25, and 33 (panels). YAP = years after planting, (**A**) Amax = maximum photosynthetic rate, (**B**) LSP = light saturation point, (**C**) Rd = dark respiration, (**D**) LCP = light compensation point, (**E**) φ = Quantum yield of photosynthesis. The color of each box plot represents a different planting density in plants per ha, red = 115; green = 128; blue = 143 (*n* = 6).

The light response curves were used to calculate the different parameters shown in Figure 4. The analyses of variance showed that the planting density did not significantly affect the various parameters. The Amax fluctuated between 6.4 µmol CO_2_ m^−2^ s^−1^. The highest values were measured in 14-year-old palms (Figure 4A). In this parameter, there were statistical differences between the age of the palms, the position of the leaf in the palm (age), and the interaction. In the palms of both ages, Leaf 17 showed consistently higher values of Amax than the others, which corroborates measurements of the maturation and metabolic stability of these leaves in the canopy. Moreover, these leaves showed the highest quantum yield of photosynthesis (Figure 4E), typical of a fully mature and active leaf.

Interestingly the light saturation point was statistically different only among the leaves. In general, LSP (Figure 4B) is related to the acclimation capacity of the plants to environmental factors [30]. At the age of the plants used in this research, the acclimation process should have happened before. It would be interesting to measure the response capacity of the plants to different stress conditions to better understand the plants’ acclimation process in terms of the photosynthetic capacity and response to light. 

The same behavior for LSP was observed in the dark respiration (Rd) (Figure 4C) and the light compensation point (LCP) (Figure 4D). There were statistical differences only between leaves. However, the most significant Rd values were measured in Leaf 1, in both palm ages. This response shows the active and accelerated metabolism of these young leaves that, in many cases, are not fully expanded and use large amounts of energy to finish their growth and development. Also, these leaves showed the most significant light compensation point due to their high dark respiration. 

### 2.4. Yield

**Figure 5:**
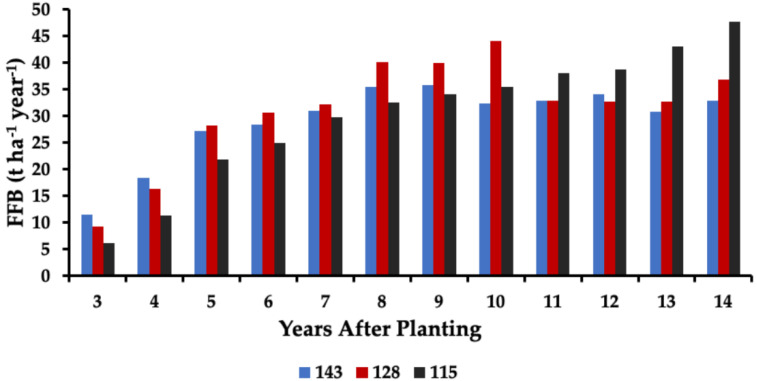
Fresh fruit bunch (FFB) production of commercial OxG plots planted at different planting densities. The FFB production was registered on an annual basis by adding the yield of all the palms in each plot. Each plot included at least 10 ha.

The interspecific OxG hybrids are recognized for their high FFB productivity [12,13], even under suboptimal conditions. The hybrids of the present study reached more than 32 t ha^−1^ year^−1^ FFB 14 years after planting, with a highest value of more than 47 t ha^−1^ year^−1^ FFB in the palms planted at 115 palms ha^−1^ (Figure 5). At 3 and 4 YAP, the most significant FFB yields were achieved with 143 palms ha^−1^, followed by 128 palms ha^−1^, with the palms planted at 115 palms ha^−1^ in the last place. At a total of 6 years after planting, FFB yield at 128 palms ha^−1^ was similar to that at 143 palms ha^−1^. However, between 8 and 10, YAP FFB yield was higher at 128 palms ha^−1^ than at 143 palms ha^−1^. After year 10, the highest FFB yields were measured at 115 palms ha^−1^, while FFB production was stabilized in the other 2 planting densities. Based on the intercepted radiation (Table 1), higher photosynthesis and, as a result, higher productivities were expected in the palms planted at 143 palms ha^−1^, which consistently showed higher interception. However, a key factor is the canopy closure, which leads to leaf overlapping and the loss of the plant’s photosynthetic capacity [17,19,21].

The cumulative FFB yield for the 14 years was 350.5 t ha^−1^ for 143 palms ha^−1^, 375.3 t ha^−1^ for 128 palms ha^−1^, and 363.2 t ha^−1^ for 115 palms ha^−1^. These results translate into 2.45 t palm^−1^ for 143 palms ha^−1^, 2.93 t palm^−1^ for 128 palms ha^−1^, and 3.16 t palm^−1^ for 115 palms ha^−1^. The palms planted at 115 palms ha^−1^ produced more FFB in the 14 years of the study, but the lower number of palms implies lower maintenance costs, which could increase the profitability of the crop [22]. 

## 3. Materials and Methods

### 3.1. Plant Material

The work was carried out in the Guaicaramo plantation in Barranca de Upía, Meta, located at a latitude of 4°26′ North, a longitude of 72°58′ West, and an altitude of 190 m above sea level. It has an average annual rainfall of 2000 to 3500 mm with a monomodal rainfall regime, an average temperature of 27 °C, and an average annual solar radiation of 465 Watts m^−2^. Nine commercial plots of interspecific OxG hybrids were used. The hybrids came from a cross between *Elaeis oleifera*, collected in the municipality of Coari (central basin of the Amazon River, north of Brazil), and pollen from African *E. guineensis pisiferas* La Mé (CIRAD). Palms of 4, 6, and 14 years of age at 9, 9.5, and 10 m planting distances, corresponding to densities of 143, 128, and 115 palms per hectare, respectively, were used. 

### 3.2. Light Interception

Radiation measurements were made from January to February (dry season). To determine the radiation interception (RI), the incident and transmitted photosynthetically active radiation (PAR) below the canopy were measured between 11:00 and 13:00 h [43] in 6 palms per age and per planting density. The *K* value depends on the sun’s horizon (α) angle, which varies with latitude, the year’s season, and through the day. To correct for the angle of the sun, *K* can be divided by sen(α), or measurements can be made near noon when sen(α) is approximately 1 [44]. 

The incident radiation (I) was measured using the SunScan Canopy Analysis System (Delta T Devices Ltd., Burwell, UK, 1996), which measures incident and transmitted PAR. SunScan consists of a beam fraction sensor (BFS) placed in non-shaded areas such as clearings in the vegetation or secondary roads adjacent to the lots, ensuring the solar radiation has an unobstructed path to the sensor. At the same time, the transmitted radiation (Io) was recorded (which is the radiation that passes through the canopy) with a 1-meter-long probe with 64 equally spaced photodiodes that recorded the transmitted radiation. 

The following equation was used for the calculation of intercepted radiation:

Intercepted Radiation = (Incident PAR − Transmitted PAR)/Incident PAR

or,

IR = 1 − [(Transmitted PAR)/(Incident PAR)](3)

The extinction coefficient for the PAR is higher than that of the total radiation. The ratio is approximately 1.4:1, respectively, and some indications show that this ratio is similar for oil palms [7].

**Figure 6:**
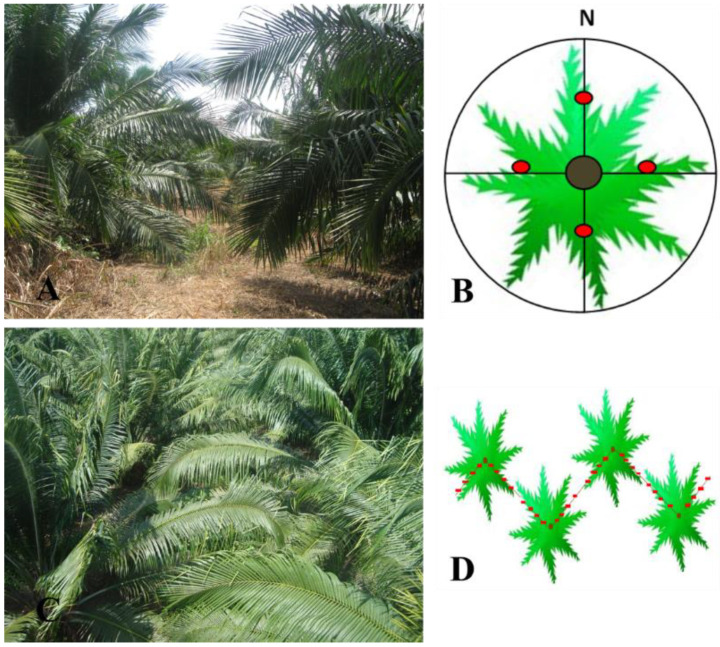
Sampling methods were used to determine intercepted solar radiation: (**A**) appearance of plots of three-year-old palms, (**B**) circular sampling system, (**C**) appearance of plots of five-year-old palms, (**D**) triangular sampling methods.

In the 4-year-old group of palms, measurements of the transmitted radiation were taken individually at 3 points per cardinal point because, at that time, the canopy was not closed, while in the 6- and 14-year-old groups of palms, the transmitted radiation was taken using the triangular method proposed by [45]. In the two methods, a total 12 sampling points per palm were taken (Figure 6).

The methodology proposed by Awal and Ishak [45] was used for estimating the leaf area (LA), and the leaf area index (LAI) was determined using the following equation:

LAI = LA × LN × PD/10,000 m^2^ (4)

where LA = leaf area (m^2^); LN = number of leaves per palm; and PD = planting density (number of palms per hectare).

With the data obtained, the Monsi and Saeki model for the continuous canopy was applied, as modified for oil palms [25], and extinction coefficients were determined for each age class and planting density. Data were subjected to regression analysis for transmitted radiation and LAI variables, and the corresponding *Kc* values were determined.

### 3.3. Photosynthetic Characterization and Yield

The actual photosynthesis was measured following the methodology of Bayona-Rodriguez and Romero [46] in Leaves 9, 17, and 25 of 10 plants that were 4, 6, and 14 years old at the indicated planting densities, for a total of 270 plants. For each plant, 2 different leaflets were measured, and the plant photosynthesis was recorded as the average photosynthesis from the 2 leaflets. The light response curves were constructed, and the photosynthetic performance parameters were calculated according to Rivera-Mendez [47] in Leaves 1, 9, 17, 25. and 33 of 3 independent palms, 6 and 14 years old, planted at the 3 planting densities. A total of 2 leaflets were used for constructing light response curves per palm, for a total of 180 curves of 90 independent palms. The photosynthetic parameters per palm corresponded to the average of the 2 leaflets.

The FFB production was recorded in every commercial plot for 12 years (14 years after planting) starting 2 years after planting. The FFB production in terms of t ha^−1^ year^−1^ was used to calculate the cumulative yield in each planting density. To estimate the yield of individual plants, the cumulative yield was divided by the number of the palms according to the planting density. The optimal planting density was calculated as previously reported [14,22].

### 3.4. Experimental Design and Statistical Analysis

A complete randomized block design under a factorial arrangement was used. The factors consisted of the age of the palms (4, 6, and 14 years after planting), planting density (115, 128, and 143 palms ha^−1^), and the leaf position in the palm (age of the leaf), with Leaves 1, 9, 17, 25, and 33 analyzed for light interception and light responses curves, and Leaves 9, 17, and 25 analyzed for actual photosynthesis. For yield, the total FFB production of the commercial plots was recorded on an annual basis. A three-way analysis of variance was used after testing for normality and variance homogeneity. All the statistical analyses were performed using R software.

## 4. Conclusions

The interspecific OxG hybrids are highly productive, with FFB yields that, in the present study, reached more than 47 t ha^−1^ year^−1^. This productivity was achieved thanks to the efficiency of the OxG hybrid to intercept the light, which in 14-year-old palms was more than 92% of the incident light in the palms under high density. In this case, LAI played a significant role in the light interception, such that the highest interception was achieved with the largest LAI (5.2) measured in 14-year-old palms at the 143 planting density. However, the highest LAI was not directly related to FFB yield because the highest yield was reached at 14 YAP in the plants at the 115 planting density. The shading and competition for resources present in the plants at the 143 and 128 palms ha^−1^ affected the photosynthetic capacity and therefore the overall productivity of the palms. The palms presented the best performance at the 115 planting density. This density at the end of the study showed the best photosynthetic parameters, the highest cumulative FFB yield, and the highest FFB per palm. Using the equation given by Corley [22], the optimum planting density for the hybrids of the present study would be 120 palms ha^−1^, corresponding to a planting distance of 9.8 m between plants.

It is important to note that the oil palm canopy closes before the leaves have reached their maximum size, unlike other crops. The results of this work show differences in the OxG hybrids’ canopy development patterns at different ages with respect to *E. guineensis*. Therefore, it is necessary to establish canopy expansion stages over time at contrasting densities and determine at which point the canopy is closed and in a stable growth stage. 

## Figures and Tables

**Table 1:** Averages of intercepted and transmitted radiation, leaf area index, and the estimated *Kc* values for each oil palm age and planting density.

Age	Density Palms ha^−1^	Leaf Number	Intercepted Radiation	Transmitted Radiation	LAI	*Kc* (Total Radiation)	*Kc* (PAR)
4	143	47	0.7606	0.2394	2.4 ± 0.111	0.42	0.58
	128	46	0.7883	0.2117	2.5 ± 0.094	0.44	0.61
	115	41	0.8303	0.1697	2.7 ± 0.061	0.46	0.64
6	143	44	0.9058	0.0841	4.3 ± 0.212	0.41	0.58
	128	43	0.8753	0.1246	3.7 ± 0.139	0.40	0.56
	115	42	0.7968	0.2031	3.0 ± 0.212	0.38	0.54
14	143	38	0.9258	0.0809	5.2 ± 0.171	0.36	0.50
	128	37	0.8942	0.1057	4.7 ± 0.303	0.35	0.49
	115	35	0.7901	0.2098	3.8 ± 0.162	0.29	0.41

## Data Availability

The data presented in this study are available on request from the corresponding author. The data are not publicly available due to privacy restrictions.
